# New insight into dolphin morbillivirus phylogeny and epidemiology in the northeast Atlantic: opportunistic study in cetaceans stranded along the Portuguese and Galician coasts

**DOI:** 10.1186/s12917-016-0795-4

**Published:** 2016-08-26

**Authors:** Maria Carolina Rocha de Medeiros Bento, Catarina Isabel Costa Simões Eira, José Vitor Vingada, Ana Luisa Marçalo, Marisa Cláudia Teixeira Ferreira, Alfredo Lopez Fernandez, Luís Manuel Morgado Tavares, Ana Isabel Simões Pereira Duarte

**Affiliations:** 1 Centre for Interdisciplinary Research in Animal Health, Faculty of Veterinary Medicine, University of Lisbon, 1300-477 Lisbon, Portugal; 2 Department of Biology and CESAM, University of Aveiro, 3810-193 Aveiro, Portugal; 3 Portuguese Wildlife Society, Department of Biology, Minho University, 4710-057 Braga, Portugal; 4 Department of Biology and CESAM, Minho University, 4710-057 Braga, Portugal; 5 Department of Biology and CBMA, Minho University, 4710-057 Braga, Portugal; 6 Coordinadora para o Estudo dos Mamíferos Mariños, 36380 Gondomar, Pontevedra Spain

**Keywords:** Cetacean morbillivirus, Dolphin morbillivirus, Striped dolphins, Eastern Atlantic

## Abstract

**Background:**

Screening Atlantic cetacean populations for Cetacean Morbillivirus (CeMV) is essential to understand the epidemiology of the disease. In Europe, Portugal and Spain have the highest cetacean stranding rates, mostly due to the vast extension of coastline. Morbillivirus infection has been associated with high morbidity and mortality in cetaceans, especially in outbreaks reported in the Mediterranean Sea. However, scarce information is available regarding this disease in cetaceans from the North-East Atlantic populations. The presence of CeMV genomic RNA was investigated by reverse transcription-quantitative PCR in samples from 279 specimens stranded along the Portuguese and Galician coastlines collected between 2004 and 2015.

**Results:**

A total of sixteen animals (*n* = 16/279, 5.7 %) were positive. The highest prevalence of DMV was registered in striped dolphins (*Stenella coeruleoalba*) (*n* = 14/69; 20.3 %), slightly higher in those collected in Galicia (*n* = 8/33; 24.2 %) than in Portugal (*n* = 6/36; 16.7 %).

**Conclusions:**

Phylogenetic analysis revealed that, despite the low genetic distances between samples, the high posterior probability (PP) values obtained strongly support the separation of the Portuguese and Galician sequences in an independent branch, separately from samples from the Mediterranean and the Canary Islands. Furthermore, evidence suggests an endemic rather than an epidemic situation in the striped dolphin populations from Portugal and Galicia, since no outbreaks have been detected and positive samples have been detected annually since 2007, indicating that this virus is actively circulating in these populations and reaching prevalence values as high as 24 % among the Galician samples tested.

**Electronic supplementary material:**

The online version of this article (doi:10.1186/s12917-016-0795-4) contains supplementary material, which is available to authorized users.

## Background

Morbillivirus infection affects mainly the upper respiratory tract, central nervous system and the immune system [1, 2] and has been identified as a cause of death and stranding in marine mammals [3]. In odontecetes, infection has been associated with high mortality rates occurring during disease outbreaks in different parts of the world [4]. The mortality rate in striped dolphins from the Mediterranean Sea in the beginning of the nineties was the highest recorded so far [2, 5, 6]. Further studies are needed to deepen the knowledge about this disease. An integrated approach taking into consideration epidemiological and environmental parameters should provide a better picture of the ecology and evolution of Cetacean Morbillivirus (CeMV) in free-ranging cetaceans [2].

CeMV includes three well characterized viral strains [7]: porpoise morbillivirus (PMV), dolphin morbillivirus (DMV) and pilot whale morbillivirus (PWMV); three novel cetacean morbillivirus strains were recently reported [8–10], adding to the genetic diversity of these viruses.

Morbilliviruses affecting cetaceans have been described in the last decades [2] after the initial detection of viral antigens in these species in the late eighties. The first evidence of morbillivirus infection in cetaceans occurred in 1988 during a PMV outbreak, when the viral antigen was detected in harbour porpoises (*Phocoena phocoena*) stranded in Ireland [11]. In the early nineties, dolphin morbillivirus (DMV) was isolated from striped dolphins from the Mediterranean [12, 13]; in 2000 PWMV was first described in a long-finned pilot whale (*Globicephala melas*) from the US coast [14] and later, in 2011, from a short-finned pilot whale (*Globicephala macrorynchus*) in the Canary Islands [15].

Due to the virus pathogenic impact on cetacean populations, further information about morbillivirus infection in cetaceans worldwide is relevant to understand its epidemiology in these animals. Studying infectious diseases in these species is important, especially considering that additional non-infectious aggressions, mainly due to human activities, render these populations even more susceptible to disease. An annual average of 200 stranded cetaceans were registered between 2010 and 2012, considering the Algarve and the Northern region of the Portuguese continental coast [16] and fisheries bycatch was identified as the most significant cause of death. To this date, no molecular data was published on morbillivirus infection in animals stranded in Portugal or northern Spain. In 2014, dolphin morbillivirus infection was reported in a retrospective study affecting striped dolphins and a common dolphin from the Canary Islands [17], causing non-suppurative meningoencephalitis. Also, a fatal systemic morbillivirus infection was detected in a bottlenose dolphin stranded in 2005 in the Canary Islands [18]. It was suggested that DMV was not endemic in harbour porpoises and common dolphins (*Delphinus delphis*) from the NE Atlantic (British Isles) in the period 1996–1999 [19], as low antibodies titres were detected in animals from Spain and the North Sea.

DMV infection apparently did not persist as an endemic infection in Mediterranean striped dolphins after the 1990–92 epidemic [19]. Both epidemics in the Mediterranean Sea (1990–92 and 2006–07) started near the Gibraltar Strait [20] and it has been suggested that DMV-infected cetaceans may have entered the Strait of Gibraltar and infected striped dolphins, the most common cetacean at the time [7, 21]. Pilot whales had been already proposed as reservoirs in 1995 [22, 23]. Later, in 2006 several long-finned pilot whales were found stranded along the coast of the Alborean Sea,and morbillivirus infection was detected [24]. In this epidemic, deaths were first detected close to the Gibraltar Strait and spread further into the Mediterranean Sea. Recently described sequences found in striped dolphins from the Canary Islands show high identity with sequences from the Mediterranean outbreaks, indicating the possible circulation of viruses between the Atlantic and the Mediterranean [17]. The role of other cetacean species as reservoirs needs to be further assessed.

The objective of the present study was to clarify not only the prevalence of DMV in cetacean populations from the eastern Atlantic, but also to investigate the relationship between the dolphin morbillivirus circulating in the eastern Atlantic and elsewhere in the world, especially in the Mediterranean.

## Methods

### Sample collection

Stranded cetaceans were collected by the Sociedade Portuguesa de Vida Selvagem (SPVS) in Northern Portugal and the Algarve within the Marine Animal Stranding Network, managed by the Instituto para a Conservação da Natureza e Florestas (ICNF) and in Galicia by the Coordinadora para o Estudo dos Mamiferos Mariños (CEMMA). Permission was issued by the National Authority (ICNF) to SPVS technicians to collect wildlife samples within the national territory according to laws n.140/99, n.49/2005, n.156-A/2013, and n.316/89. Also, SPVS is a registered CITES scientific research institution (code PT009). CEMMA holds a permit from the Conselleria de Medio Ambiente, Territorio e Infraestruturas de Xunta de Galicia (Spain) to collect and maintain cetacean samples according to law 42/2007 and law 9/2001.

The animals were assigned a decomposition code (1 to 5) according to already established protocols [25]. Animals with a score ranging from to 1 to 3 (fresh to moderate decomposition) were surveyed in the present study. During necropsy, tissue samples were collected from 279 cetaceans: brain, lung, pulmonary lymph node, mesenteric lymph node, spleen, kidney and liver, whenever possible. For animals from Galicia the only available sample was the lung. Samples collected in Portugal were stored in vials with RNAlater® at −20 °C and samples collected in Galicia were frozen at −20 °C. All samples were kept in the marine animals’ tissue banks (MATBs) of SPVS and CEMMA. Samples from different species were collected between 2004 and 2015: common dolphins (DD), striped dolphins (SC), bottlenose dolphins (*Tursiops truncatus*; TT), long-finned pilot whales (*Globicephala melas*; GM), Pigmy Sperm Whale (*Kogia breviceps*; KB), True’s Beaked Whale (*Mesoplodon mirus*; MMi) and Fin whale (*Balaenoptera physalus*; BP) (Table 1). Samples were identified with a code composed by the species identification (e.g., DD, SC, TT), a number attributed to each stranding, and the year of stranding. From cetaceans stranded in the Portuguese coastline 91 animals from 2011, 56 from 2012, 33 from 2013, 49 from 2014 and 7 from 2015 were tested. From Galicia, a total of 33 lung samples from striped dolphins were tested. Available tissue samples from 10 animals stranded in Portugal from previous years were also included in this study (6 striped dolphins and 4 pilot whales from 2004 to 2009).

**Table 1:** Number of stranded cetaceans tested for DMV *per* year

		2004	2005	2006	2007	2008	2009	2010	2011	2012	2013	2014	2015	Total
Portugal	Common dolphin (DD)	–	–	–	–	–	–	–	84	45	29	29	6	193
	Striped dolphin (SC)	1	–	1	4	–	–	–	5	6	3	16	–	36
	Pilot whale (GM)	–	–	–	–	2	2	–	–	1	–	–	–	5
	Bottlenose dolphin (TT)	–	–	–	–	–	–	–	1	2	1	3	–	7
	True’s beaked whale (MMi)	–	–	–	–	–	–	–	1	–	–	–	–	1
	Pigmy sperm whale (KB)	–	–	–	–	–	–	–	–	1	–	1	–	2
	Fin whale (BP)	–	–	–	–	–	–	–	–	1	–	–	1	2
Galicia	Striped dolphin (SC)	2	1	1	2	3	6	4	2	3	5	4		33

### Total RNA extraction

Total RNA was extracted from a pool of tissue homogenates using RNeasy mini kit (Qiagen, GmbH, Germany), according to the manufacturer’s instructions. The pool included, whenever possible: lung, brain, pulmonary lymph node and mesenteric lymph node. Total RNA quantification and purity was determined using a Nanodrop 2000C spectrophotometer (ThermoScientific, USA) and stored at −80 °C until used.

### Detection of dolphin morbillivirus genomic RNA by reverse transcription-quantitative PCR (RT-qPCR)

The detection of viral RNA for the DMV strain of CeMV was performed by RT-qPCR in a StepOnePlus thermocycler (Applied Biosystems), using primers (Stabvida genomics lab, Portugal) and probe (Eurogentec), targeting the N gene of DMV, as previously described [26] (Table 2). A previously detected positive sample for DMV was used as a positive control of the PCR reaction. Negative reaction controls were always included.

**Table 2:** Primers and probe set used in RT-qPCR assays

	5′ Fluorophore	3′ Quencher	Sequences (5′–3′)	Amplicon size	Annealing (°C)	
DMV-N-FP	–	–	TGCCAGTACTCCAGGGAACATCCTTC	173	60	[26]
DMV-N-RP	–	–	TTGGGTCGTCAGTGTTGTCGGACCGTT	173	60	
DMV-N-probe	Cy3	BHQ1	A + CA + CCAAA + AGGGA + CA	–	60	

**Fig. 1:**
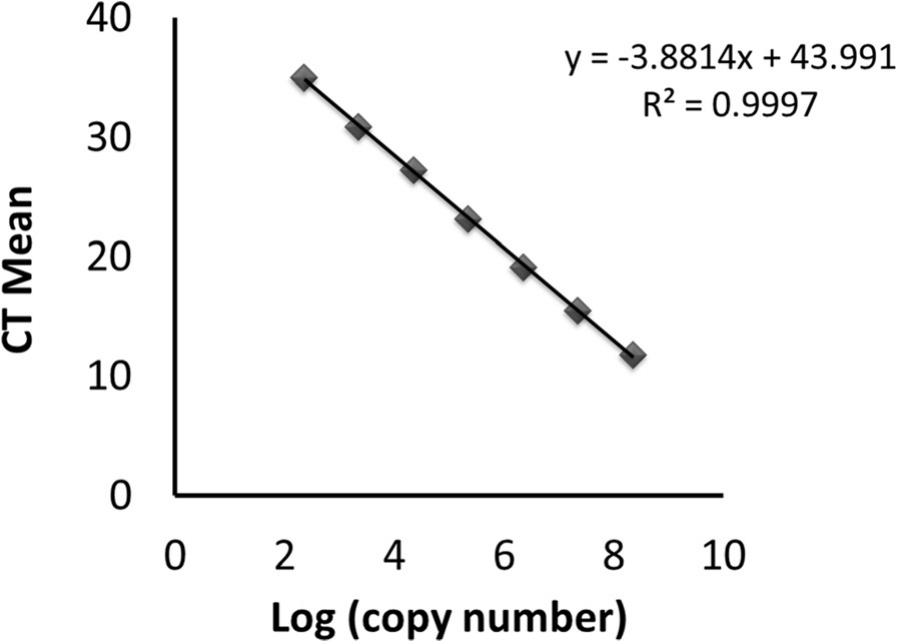
Standard curve and equation for the determination of the efficiency of the RT-qPCR for the molecular detection of DMV. The N gene fragment obtained in the RT-qPCR reaction was cloned into a plasmid vector (Pgem Teasy – Promega) and serial tenfold dilutions of the recombinant plasmid DNA were amplified by qPCR in duplicate reactions and used to construct the standard curve. *Y axis* represents the mean CT values obtained from the duplicates and *X axis* represents the LOG10 of calculated copy numbers (ranging from 2.24E + 08 to 224 copies). Calculated efficiency of 81 % was determined using the formula: Efficiency = 10(-1/slope) x (-1). Results showed a high correlation (*R*2 = 0.997)

One step RT-qPCR assays were performed using 100 ng of the template RNA, in a total reaction volume of 20 μL containing: 10 μL of 1-step qPCR-ROX Mix (2×); 1 μL of RT enhancer; 0,2 μL of Verso Enzyme Mix (Verso 1-Step qRT-PCR ROX kit, ThermoScientific®); 0.4 μM of each primer and 0.25 μM of probe. For positive samples, total RNA was extracted individually for each of the available organs and the infection was evaluated individually in the different organs. The amplified DMV fragment was cloned into a plasmid vector (Pgem Teasy – Promega) and serial tenfold dilutions of the recombinant plasmid DNA were used to construct the standard curve (Fig. 1). The results showed a high correlation (*R*^2^ = 0.997) with a calculated efficiency of 81 %. The primers and probe could detect viral RNA copies down to 10^2^, and the limit of detection was 224 copies.

### Conventional PCR for amplification of DMV genes

**Fig. 2:**
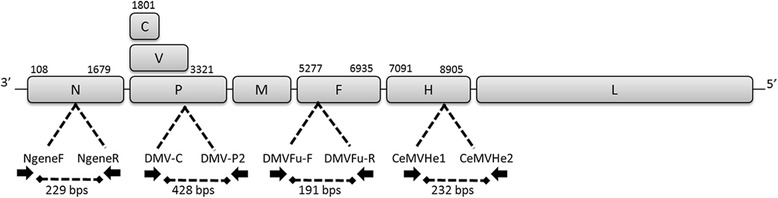
Schematic representation of the primers used to amplifiy different genomic regions by conventional RT-PCR. Schematic representation of the DMV genome and location of the primers used in the conventional RT-PCR reactions, targeting: the N gene (NgeneF and NgeneR) to amplify a fragment of 229 basepairs (bps); the P gene (DMV-C and DMV-P2) for a fragment of 428 bps; the F gene (DMVFu-F and DMVFu-R) (191 bps) and for a fragment of 232 bps from the H gene (CeMVHe1 and CeMVHe2)

Additional sequences were amplified from the positive samples by conventional reverse transcription-PCR (RT-PCR) using previously described primers (Table 3) purchased from Stabvida genomics lab (Portugal). Primers were used in different combinations, targeting different genomic regions (Fig. 2).

**Table 3:** Primers used in conventional PCR assays

Primer	Target gene	Sequence (5′–3′) (sense)	Tm	Genome position	Reference
CeMV-He1	H	CRTTGATACTYGTGGGTGTG (+)	59	7194–7213	[15]
CeMV-He2	H	TGTTAACTTCTGGGGCATCC (−)	59	7407–7426	
DMVFu-F	F	GGCACCATAATTAGCCAGGA (+)	51	6483–6502	
DMVFu-R	F	GCCCAGATTTGTGCCTACAT (−)	51	6655–6674	
DMV-C	P	ATGTTTATGATCACAGCGGT (+)	51	2132–2151	[35]
DMV-P2	P	ATTGGGTTGCACCACTTGTC (−)	51	2541–2560	
NgeneF	N	CCHAGRATYGCTGAAATGATHTGTGA (+)	48	849–874	[14]
NgeneR	N	AACTTGTTCTGRATWGAGTTYTC (−)	48	1056–1078

The obtained amplicons were used to perform a phylogenetic analysis of the DMV sequences, along with sequences retrieved from NCBI for the same genes. L and M genes were not targeted in the conventional RT-PCR since very few sequences were available at the NCBI database.

The amplicons were directly sequenced by Sanger sequencing at Stabvida, Portugal and the specificity of the nucleotide sequences was compared by Blast analysis http://blast.ncbi.nlm.nih.gov/Blast.cgi with CeMV sequences available in the GenBank.

### Phylogenetic analysis

The nucleotide sequences of the Portuguese and Galician sequence datasets available in the GenBank (National Center for Biotechnology Information) repository,with the following accession numbers KP835987; KP835991; KP835995; KP835999; KP836003; KP835986; KP835990; KP835994; KP835997; KP836002; KP836006; KP835985; KP835989; KP835993; KP835996; KP836001; KP836005; KP835984; KP835988; KP835992; KP835998; KP836000; KP836004; KP835983; KT878649; KT878650; KT878651; KT878652; KT878653; KT878654; KT878655; KT878656; KT878657; KT878658; KT878659; KT878660; KT878661, were compared with the available CeMV sequences and outgroup taxa (Canine Distemper Virus [CDV], Phocine Distemper Virus [PDV] and Measles Virus [MV]), retrieved from GenBank (Table 4), according to their primary structure similarity using the multiple alignment ClustalW program [27].

**Table 4:** Accession number for GenBank sequences used to the phylogenetic analysis and corresponding description

Complete genomes and common sequences	NC_0014981	Measles Virus	AY649446	Canine Distemper Virus
	KC802221	Phocine Distemper Virus	AJ608288	Dolphin Morbillivirus complete genome
	HQ829973	Striped dolphin 2007 SP (Med)	HQ829972	Long-finned pilot whale 2007 SP (Med)
Gene N	X84739	Porpoise 1988 IRL	AF200818	Long-finned pilot whale 1999 USA
	FJ842380	Short-finned pilot whale 1996 SP (Can Isl)		
Gene P	KF695110	Bottlenose dolphin 2005 SP (Can Isl)	JX195718	Longman’s beaked whale 2010 USA
	EU039963	Long-finned pilot whale 2007 SP	KF650727	Porpoise 1990 NL
	EF451565	White-beaked dolphin 2007 GM	AF200817	Long-finned pilot whale 1999 USA
	AF333347	Pigmy sperm whale 2001 TW	KJ139451	Striped dolphin 2002 SP (Can Isl)
	KJ139452	Striped dolphin 2007 SP (Can Isl)	JN210891	Striped dolphin 2011 SP (Med)
	KF711855	Guiana dolphin 2010 BR	KJ139454	Striped dolphin 2011 SP (Can Isl)
	KC572861	Striped dolphin 2012 SP (Med)	KJ139453	Striped dolphin 2009 SP (Can Isl)
	KR337460	Fin whale 2013 IT	KR704575	Longman’s beaked whale 2013 NC
	KC888945	White-beaked dolphin 2011 NL		
Gene F	AJ224704	Striped dolphin 90’s SP	Z30086	DMV 1994
	FJ842382	Short-finned pilot whale 1996 SP		
Gene H	FJ648457	Porpoise MV 1988 IRL	AJ224705	Striped dolphin 90’s SP
	Z36978	DMV 1994	FJ842382	Short-finned pilot whale 1996 SP (Can Isl)

*SP* Spain, (*MED*) Mediterranean, (*Can Isl*) Canary Islands, *IRL* Ireland, *USA* United States of America, *NL* Netherlands, *GM* Germany, *TW* Taiwan, *BR* Brazil, *IT* Italy, *NC* New Caledonia

Six sets of alignments were considered for the phylogenetic analysis: nucleotide sequence alignments for genes N, P, F and H, composed by sequences of 218, 342, 449 and 316 base pairs, respectively; concatenated sequence of amino acids (540 aa) and nucleotides (1446 bps). Due to heterogeneity of the available DMV sequences it was not feasible to maintain the same set of DMV sequences in the alignment for each gene. In the concatenated alignment only the sequences with all partial genomic regions were included. The multiple sequence alignments were manually corrected with Jalview, Version 2.0.1 [28] removing long internal gaps and unmatched ends to maximize genetic similarities and phylogenetic trees were inferred by Bayesian methods (MrBayes v.3.2.1) [29, 30].

For the Bayesian analysis a Markov chain Monte Carlo (mcmc) simulation technique was carried out to approximate the PP of trees [30]. The evolutionary GTR (nucleotides) and LG (amino acids) models were selected with gamma-distributed rate variation across sites and a proportion of invariable sites (rates = invgamma). The analysis was initiated using a random tree from the dataset with four chains running simultaneously for 20 × 10^6^ generations, sampling every 100 generations. The first 25 % trees were discarded and a majority rule consensus tree was generated from the remaining trees.

The graphical representation and edition of the phylogenetic tree were performed with FigTree v1.3.1. Only support values equal or greater than 0.70 of PP are shown in the trees.

### Statistical analysis

Chi-square test of association was performed to assess if the difference in prevalence was statistically significant between different species (DD and SC) and between animals from different origins (Portugal and Galicia). For this analysis an online website for statistical computation was used url: http://vassarstats.net/. A confidence interval (CI) of 95 % (for a *p* value ≤0.05) was considered for all the statistical analysis.

## Results

A total of 16 DMV positive cetaceans were identified by RT-qPCR, representing a prevalence of 5.7 % (IC95 %: 3.42;9.32). With respect to the Portuguese coastline, 8 positive animals were detected, including 6 striped dolphins (SC) and 2 common dolphins (DD) [SC/15/2007, SC/257/2011, SC/221/2012, DD/302/2012, SC/11/2013, DD/191/2013, SC/193/2014 and SC/290/2014]. In Galicia, 8 positive striped dolphins were detected [SC/21/2007, SC/24/2008, SC/31/2009, SC/42/2010, SC/49/2011, SC/51/2012, SC/53/2012 and SC/55/2012]. Among all cetacean species, striped dolphins (*n* = 69) revealed a significantly higher DMV prevalence reaching 20.3 % (IC95 %: 11.92; 32.02), whereas common dolphins (*n* = 139) recorded a prevalence of 1.0 % (IC95 %: 0.18; 4.09) (*P* value *0.00*). Positive striped dolphins were detected every year (from 2007 to 2014) while positive common dolphins were only detected in 2012 and 2013. The DMV prevalence in striped dolphins stranded in Galicia was 24.2 % (IC95 %: 11.74; 42.63) whereas in Portugal the DMV prevalence was 16.7 % (IC95 %: 6.97; 33.47). From the positive animals stranded along the Portuguese coastline, each organ included in the tissue pool was tested individually for viral RNA. Two animals tested positive in all available organs; four were positive for viral RNA only in the brain and one animal tested positive in the lung, and in the pulmonary and mesenteric lymph node (Table 5). Lung was the only available sample to test in samples from Galicia.

**Table 5:**
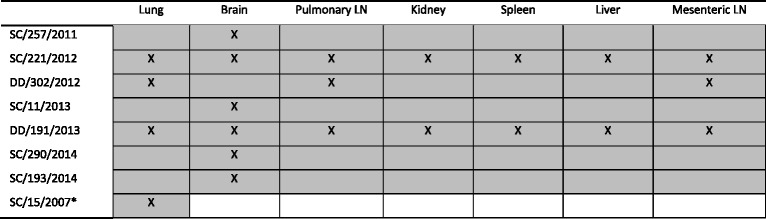
Mapping of DMV infection in the available organs in Portuguese samples

Tested organs are shown in grey and positive organs are marked with an (X)

* only lung samples were available

For samples SC/15/2007, SC/257/2011, SC/53/2012, SC/55/2012, DD/302/2012, SC/290/2014, SC/11/2013, SC/31/2009, SC/51/2012, SC/21/2007, SC/221/2012 and DD/191/2013 longer genomic regions were amplified by one step RT-conventional PCR with the primers described previously (Table 3). For samples SC/24/2008, SC/42/2010, SC/49/2011 and SC/193/2014 no fragments were amplified by conventional RT-PCR.

For the nucleotide sequences of each genomic region, phylogenetic trees were inferred by Bayesian methods. All trees exhibited a similar sequence topology, supported by robust PP values, regardless of the total number of sequences in each tree.

**Fig. 3:**
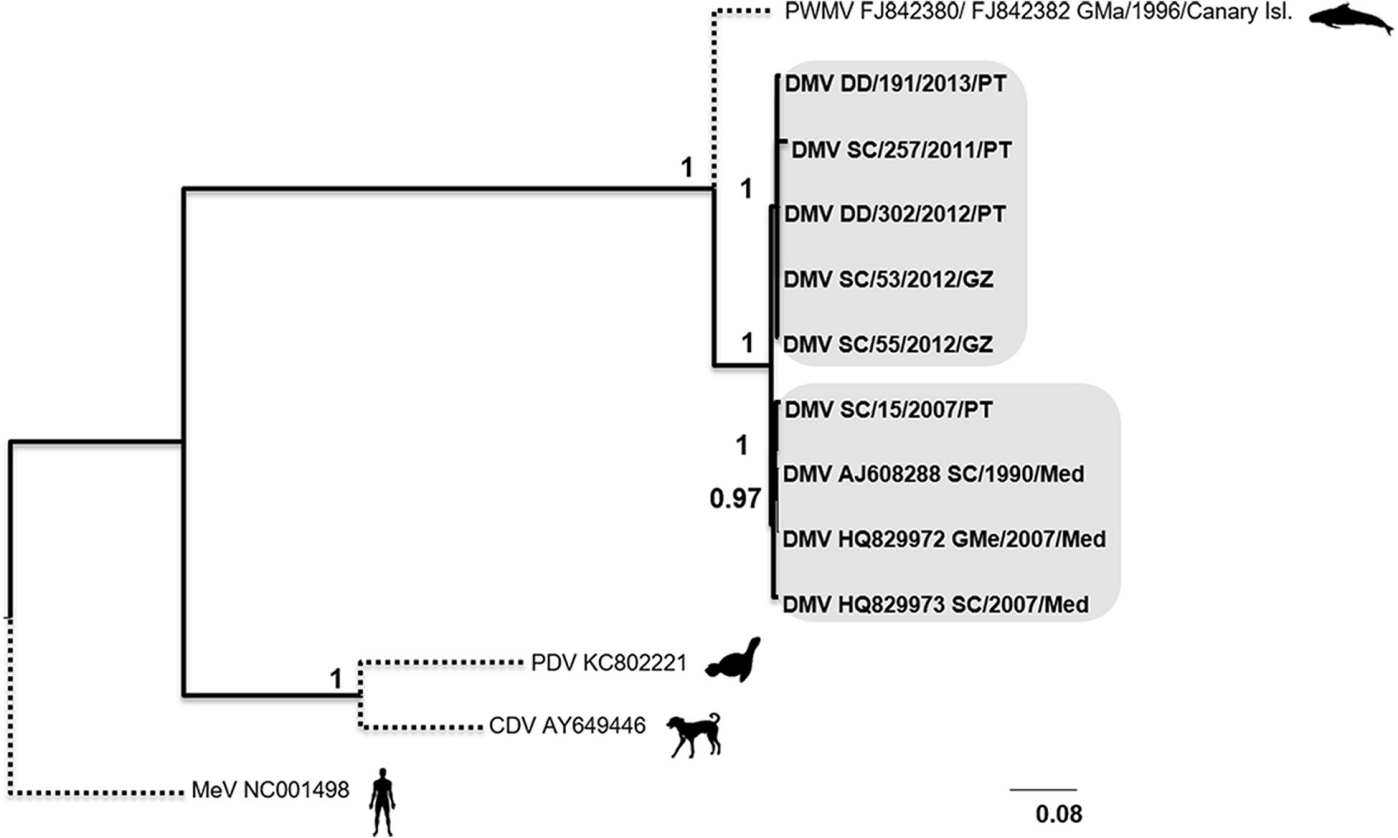
Phylogenetic tree for the concatenated nucleotide sequences. Phylogenetic tree generated with concatenated nucleotide sequences alignment, inferred by Bayesian methods. Sequences for the outgroup taxa were retrieved from NCBI for Pilot Whale Morbillivirus (PWMV [accession numbers FJ842380 and FJ842382]); Phocine Distemper Virus (PDV [accession number KC802221]); Canine Distemper Virus (CDV [accession number AY649446]) and Measles Virus (MV [accession number NC001498]). Three DMV sequences were also retrieved from NCBI: one isolate from a pilot whale [HQ829972] and one from a striped dolphin [HQ829973], both from 2007; one isolate from 1990, also from a striped dolphin [AJ608288]. Sequences obtained for animals Sc/257/2011 (KP835983; KP835984; KP835985; KP835986), Dd/191/2013 (KP836003; KP836004; KP836005; KP836006), Sc/53/2012 (KP835991; KP835992; KP835993; KP835994), Dd/302/2012 (KP835999; KP836000; KP836001; KP836002) and Sc/55/2012 (KP835987; KP835988; KP835989; KP835990), Sc/15/2007 (KP835995; KP835996; KP835997; KP835998) were also included in this tree

**Fig. 4:**
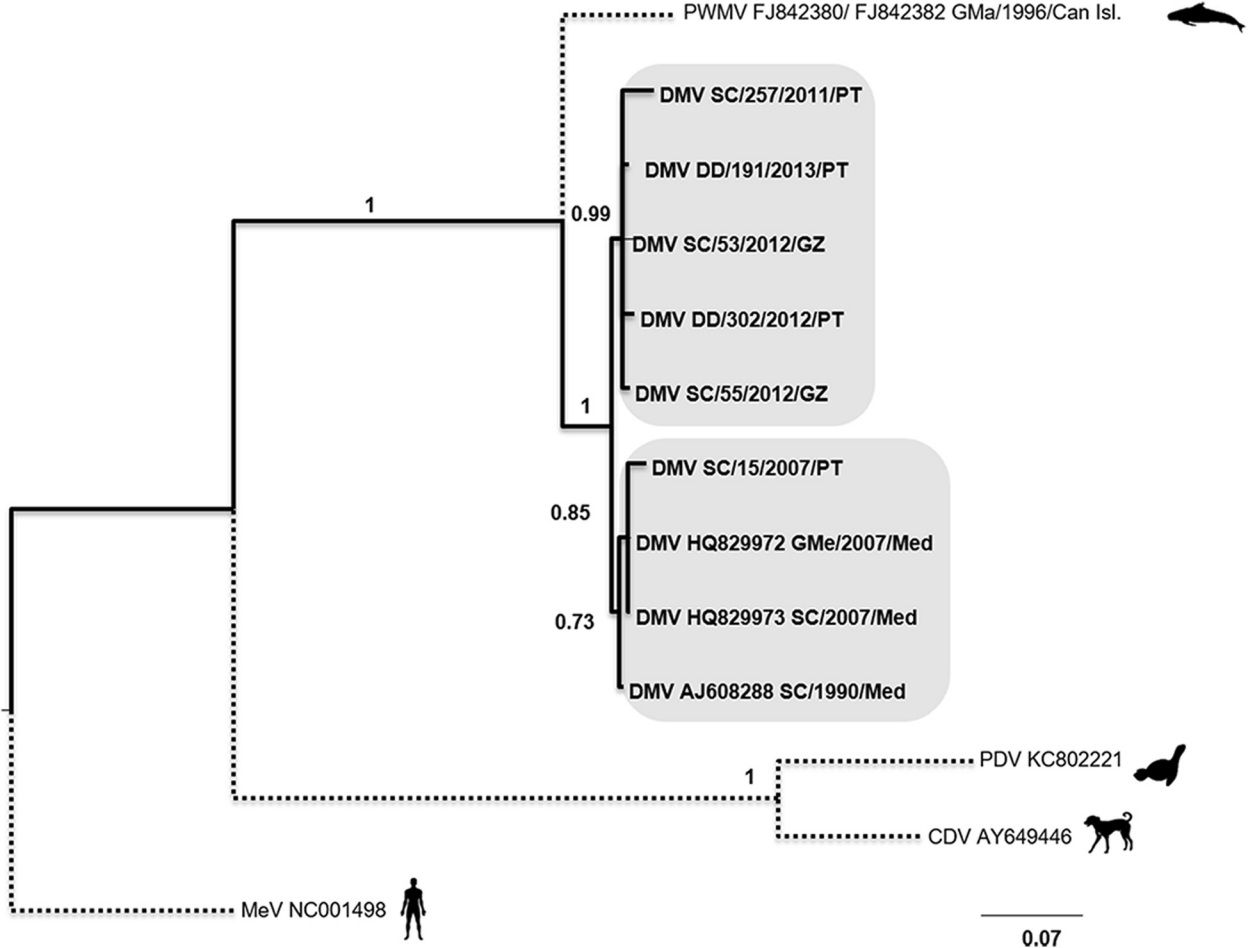
Phylogenetic tree for the concatenated amino acid sequences. Phylogenetic tree generated with the concatenated amino acid sequences alignment, inferred by Bayesian methods. Sequences for the outgroup taxa were retrieved from NCBI for Pilot Whale Morbillivirus (PWMV [accession numbers FJ842380 and FJ842382]); Phocine Distemper Virus (PDV [accession number KC802221]); Canine Distemper Virus (CDV [accession number AY649446]) and Measles Virus (MV [accession number NC001498]). Three DMV sequences were also retrieved from NCBI: one isolate from a pilot whale [HQ829972] and one from a striped dolphin [HQ829973], both from 2007; one isolate from 1990, also from a striped dolphin [AJ608288]. Sequences obtained for animals Sc/257/2011 (KP835983; KP835984; KP835985; KP835986), Dd/191/2013 (KP836003; KP836004; KP836005; KP836006), Sc/53/2012 (KP835991; KP835992; KP835993; KP835994), Dd/302/2012 (KP835999; KP836000; KP836001; KP836002) and Sc/55/2012 (KP835987; KP835988; KP835989; KP835990), Sc/15/2007 (KP835995; KP835996; KP835997; KP835998) were also included in this tree

In the tree of the concatenated nucleotide sequences (Fig. 3) the CeMV sequences were distributed in three main branches supported by high PP values. Portuguese and Galician samples from 2011, 2012 and 2013 were included in one branch; sequences from the Mediterranean from 2007, early nineties (AJ608288) and the Portuguese sequence SC/15/2007 in another branch. The only PWMV included in this tree is isolated in a third branch. The tree of the amino acid concatenated sequences presented a similar pattern (Fig. 4), although with a rearrangement within the older sequences branch ([SC/15/2007, GM/2007, Med, SC/2007/Med]; [SC/1990/Med]).

In the nucleotide tree for the F gene (Additional file 1) additional available sequences from the early nineties were included. Samples collected in the Atlantic during the 2011-2013 period clustered in the same branch; samples from the nineties clustered in a separate branch, and samples from the Mediterranean from 2007 clustered in a third branch, together with the sample SC/15/2007, similarly to the distribution of the concatenated trees. The PWMV was included in a unique branch. All branches were supported with a high PP values.

For the H gene nucleotide tree (Additional file 2), a higher number of sequences were included. A set of sequences (9) from Portugal and Galicia ranging from 2009 to 2014 clustered in the same branch, supported by a PP value of 0.98. The SC/15/2007 sequence still clustered with Mediterranean samples from 2007 and samples from the early nineties were grouped in a separate branch. The new sequence for PMV included in this tree, branches out from the DMV samples, similarly to the PWMV sequence (PP value of 0.9).

**Fig. 5:**
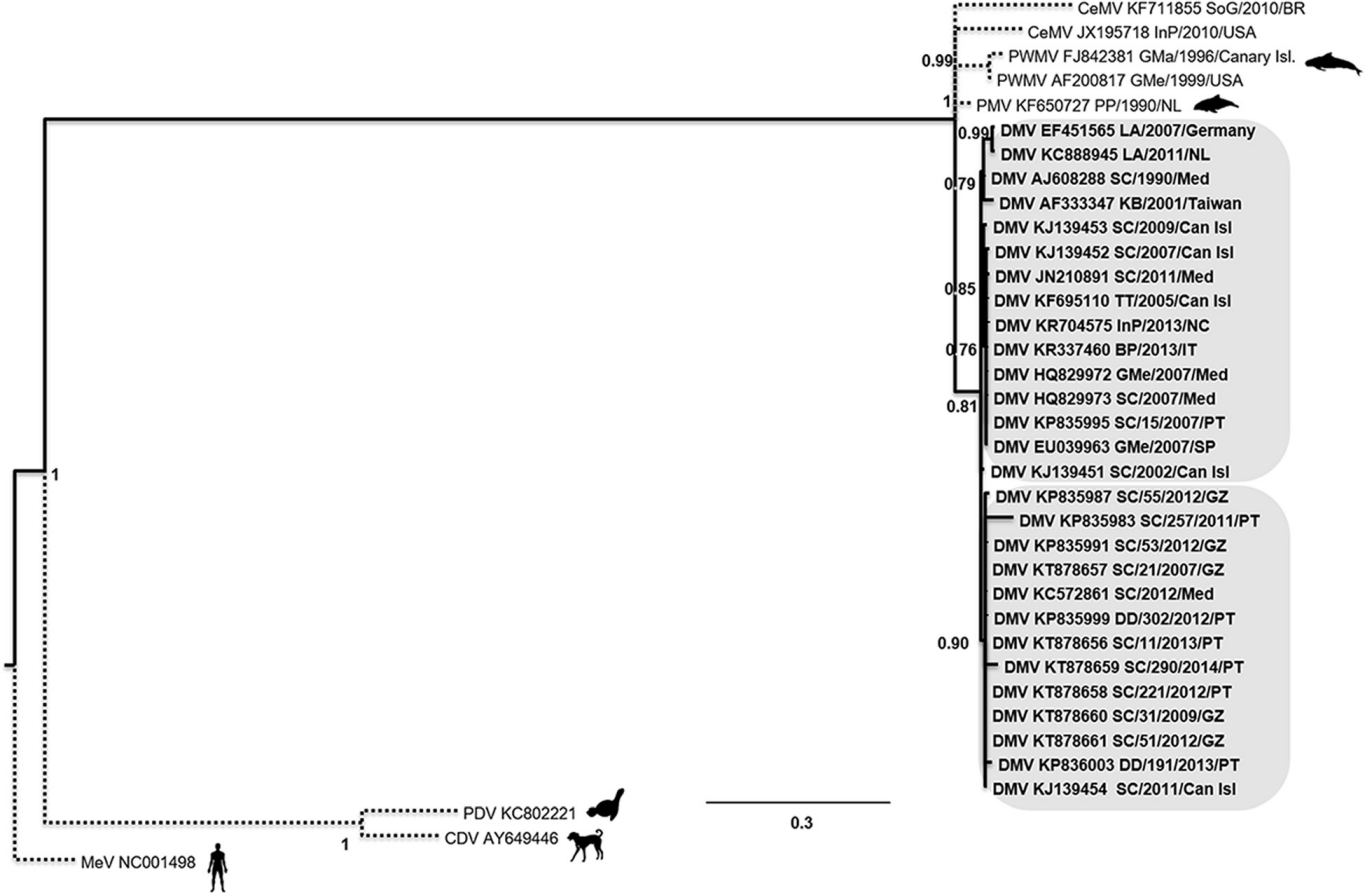
Phylogenetic tree for the P gene nucleotidic sequences. Phylogenetic tree generated with the aligned sequences for the P gene, inferred by Bayesian methods. Sequences for the outgroup taxa were retrieved from NCBI for Pilot Whale Morbillivirus (PWMV [accession numbers FJ842380 and AF200817]); Porpoise Morbillivirus (PMV [accession number FJ650727]); Phocine Distemper Virus (PDV [accession number KC802221]); Canine Distemper Virus (CDV [accession number AY649446]) and Measles Virus (MV [accession number NC001498]). Two recently described sequences of CeMV (JX195718 and KF711855) were also included, along with: one isolate of DMV from 2002 collected in the Canary Islands (KJ139451), one collected in Taiwan in 2001 (AF333347), five sequences from 2007 collected in Spain and Germany (EF451565, HQ829972, HQ829973, EU039963, KJ139452), one sequence from the 90’s (AJ608288), one from 2005 (KF695110) and one from 2011 collected in the Mediterranean (JN210891). Sequences obtained for the P gene of Portuguese and Galician isolates were also included for animals Sc/290/2014 (KT878659), Sc/11/2013 (KT878656), Sc/31/2009 (KT878660), Dd/191/2013 (KP836003), Sc/55/2012 (KP835987), Sc/51/2012 (KT878661), Dd/302/2012 (KP835999), Sc/21/2007 (KT878657), Sc/221/2012 (KT878658), Sc/257/2011 (KP835983), Sc/53/2012 (KP835991), and Sc/15/2007 (KP835995)

The nucleotide tree for the P gene (Fig. 5) contained the higher number of sequences (35). One sequence from a guiana dolphin (*Sotalia guianensis*) collected in 2010 in Brazil appeared to be a distinct strain from the already characterized strains of CeMV (PMV, PWMV and DMV. The two PWMV samples clustered in the same branch and the only PMV included in the tree was isolated from all the other sequences. All these strains were supported by high PP values. The DMV sequences included in this tree were all similar, including sequences from distinct geographic origins, such as Germany, Taiwan or the Mediterranean. Two sequences obtained from white-beaked dolphins in Germany and the Netherlands in different years (2007 and 2011 respectively) clustered together with a PP value of 0.99. Sequence AJ608288 from a striped dolphin collected in 1990 in the Mediterranean and sequence AF333347 from a pigmy sperm whale from Taiwan collected in 2001 also clustered together (PP 0.79). Samples from the Canary Islands collected in 2005, 2007 and 2009 clustered with samples from the Mediterranean (2007 and 2011), one sample from New Caledonia and one sample from Portugal (SC/15/2007). The remaining Portuguese and Galician samples clustered in the same clade with two samples with a different origin (KJ139454, Canary Islands and KC572861, Mediterranean).

The N gene nucleotide tree (Additional file 3) showed a dislocation of sequences between branches. One branch included Atlantic samples from 2011 to 2013 grouped with the SC/15/2007 sequence and with sequences from the Mediterranean (1990 and 2007); Atlantic sequences also from 2012 to 2014, were grouped separately. The remaining PWMV and PMV sequences appeared as two different outgroups.

## Discussion

In this study we surveyed 279 animals and our results indicate a higher prevalence of DMV among stranded striped dolphins (20.6 %) when compared to stranded common dolphins (1 %) from the Atlantic based populations. Similar results had been previously described in the Mediterranean during the 1990–92 and 2006–08 CeMV breakouts, when striped dolphins presented higher death and stranding rates than other species [2, 31, 32]. Several theories have been hypothesized for this higher mortality rate amongst striped dolphins in the Mediterranean: they were the most numerous species in the Mediterranean and serological studies suggested that, prior to the 2006–08 outbreak, antibody levels were low in this population rendering them more susceptible to the CeMV infection [19]; also, the fact that they are highly gregarious and tend to live in large pods could contribute to the spread of CeMV infection [33]; high polychlorinated biphenyl (PCB) levels were also detected in the affected animals, leading to the hypothesis that an impaired immune system might have facilitated the infection by CeMV; finally, genetic susceptibility as a result of inbreeding in the Mediterranean population [33], which had already been reported as relatively isolated from the Atlantic populations [34].

Prevalence among striped dolphins from Galicia was 24.2 % while prevalence in striped dolphins stranded in Portugal was 16.7 %. Although this difference was not statistically significant, it is important to highlight that prevalence among striped dolphin samples from Galicia was probably underestimated since only lung samples were tested. Samples from the Portuguese coastline allowed testing several organs and antigen was only detected in brain samples of four individuals out of the 6 positive striped dolphins. It is therefore possible that the prevalence in striped dolphins from Galicia is being strongly underestimated. Previous studies from the Atlantic based populations were performed in the western part of the Atlantic, along the USA coast, and bottlenose dolphins were the most affected cetaceans in that area. In the Canary Islands a retrospective study was published in 2014 and 6 animals were positive for CeMV (5 striped dolphins and 1 common dolphin) [17]. In this study striped dolphins seem to be the most affected species sampled from the East Atlantic.

In four animals it was not possible to amplify viral genomic fragments by conventional RT-PCR. These samples recorded high CT values in the RT-qPCR, corresponding to a low target copy number (ranging from 105 to 943 copies), which would present a downside using a less sensitive conventional assay. Also, three of the four samples were collected in animals from Galicia originally stored at −20 °C, which may possibly imply RNA degradation hampering the amplification of longer genomic fragments, by conventional RT-PCR.

The genetic distances between samples were low among all sequences included in the phylogenetic trees. Nonetheless, PP values were high and consistent in all trees particularly in the DNA concatenated tree, adding robustness to the phylogenetic arrangement.

In the phylogenetic trees for the concatenated nucleotides the grouping of viral sequences followed a temporal arrangement, with samples collected since 2007 forming different clades. When a higher number of sequences was added to the trees (P gene tree) a phylogeographic arrangement becomes clear: all samples from Portugal and Galicia cluster together (with isolates ranging from 2007 to 2014), further away from the samples from the Mediterranean. The only exception seems to be the sequence from the animal SC/15/2007,clustering with samples from the Mediterranean, as well as with samples from the Canary Islands. Even samples from animals stranded in the south of Portugal (Algarve), such as SC/11/2013, clustered separately from samples obtained in the Mediterranean. This suggests that these populations may be relatively isolated from each other, which is supported by previous findings by other authors [34]. It is worth noticing that only one sample from a striped dolphin collected in the Canary Islands clusters closer to the Portuguese and Galician samples. All the other samples from the Canary Islands are closer to Mediterranean samples.

Positive samples for DMV antigen were detected annually since 2007 to 2013, showing that the virus is circulating in cetacean populations from the Atlantic off the coast of Portugal and northern Spain and both striped dolphins and common dolphins were found to be positive to viral infection. The infection was mapped in the available organs and positive lung samples were detected without association to higher mortality or stranding rates. Further studies would be necessary to determine if these animals had an acute, sub-acute or chronic infection and if the DMV infection was the cause of death. Animals DD/191/2013, DD/302/2012, SC/21/2007, SC/51/2012, SC/53/2012, SC/11/2013 and SC/257/2011 stranded alive and were in general emaciated and with high parasite loads, suggesting a sub-acute or chronic systemic infection. Histological and immunohistochemical studies should be performed to further characterize the necropsy findings. Four animals (SC) were positive only in brain samples, which might imply the development of chronic localized encephalitis after a systemic infection.

The two common dolphins positive for viral antigen (DD/302/2012 and DD/191/2013) were both alive at the time of stranding and presented high parasite loads and poor body condition. Animals DD/302/2012 and SC/221/2012 are also positive for cetacean gamma herpesvirus (unpublished observations Bento, C.) with viral antigen detected systemically. Unlike Mediterranean populations of striped dolphin [2], morbillivirus infection seems to be endemic in the population of striped dolphins from the Atlantic. This correlates to the serological survey conducted in 2011 in which 21.6 % (*n* = 37) of the analysed cetaceans cross reacted with Canine Distemper Virus antigen in a commercially available ELISA kit (unpublished observations Bento, C.). To date, the harbour porpoise was reported as the most affected species with morbillivirus infection in the north-eastern Atlantic, although infection is probably not endemic considering porpoises’ solitary behaviour [2]. Large populations are needed to maintain morbillivirus infections as endemic [19] and although striped dolphin abundance has increased over the last years in the Portuguese Continental coast it is still a rather small population if compared to the common dolphin population (Araújo, H. personal communication). Notwithstanding, evidence suggests an endemic situation rather than an epidemic, since no outbreaks have been detected in the striped dolphin population of the Atlantic. Moreover, positive samples have been detected annually since 2007, indicating that this virus is actively circulating in this population reaching prevalence values as high as 24 % in the Galician samples. In 1999, dolphins stranded along the Atlantic coast of Spain had low antibody titres for CeMV. Considering the results obtained in this study, further serological studies are needed to deepen the knowledge about the epidemiology of this disease in striped dolphins.

Unlike striped dolphins, the prevalence of stranded common dolphins positive for viral antigen is much lower (1 %). The difference in CeMV prevalence between stranded common and striped dolphins needs to be fully assessed and further studies are needed to clarify the virus impact on cetacean populations and why do striped dolphins appear to be more susceptible to DMV infection. New approaches should be considered: viral enrichment and random amplification techniques associated with next generation sequencing could contribute to deepen the knowledge on this virus and its interaction with other pathogens.

Surveys are a unique tool to provide information on viral epidemiology, especially in free-ranging cetaceans.

## Conclusion

Our results suggest that DMV infection is endemic in striped dolphin populations of the Eastern Atlantic. Since it was first reported in cetaceans in the early nineties subtle but consistent changes in the reported viral sequences suggest that the Atlantic and the Mediterranean populations are relatively isolated from each other, as suggested by other authors. The prevalence of infection in stranded common dolphins is very low when compared to striped dolphins, and our results are in agreement with previous reports that point to a higher susceptibility of striped dolphins to CeMV. Reasons for differences in susceptibility to this viral infection in different species should be further investigated and serological surveys should also be performed to assess their protection level towards CeMV infection.

## Additional files

Additional file 1: Phylogenetic tree for the F gene nucleotidic sequences. Phylogenetic tree generated with the aligned sequences for the F gene, inferred by Bayesian methods. Sequences for the outgroup taxa were retrieved from NCBI for Pilot Whale Morbillivirus (PWMV [accession number FJ842382]); Phocine Distemper Virus (PDV [accession number KC802221]); Canine Distemper Virus (CDV [accession number AY649446]) and Measles Virus (MV [accession number NC001498]). Three DMV sequences from the 90’s were retrieved from NCBI and included in the trees (Z30086; AJ224704 and AJ608288) along with two sequences from 2007 (HQ829972 and HQ829973). Sequences obtained for the F gene of Portuguese and Galician isolates were also included for animals Sc/257/2011 (KP835986), Dd/302/2012 (KP836002), Dd/191/2013 (KP836006), Sc/53/2012 (KP835994), Sc/55/2012 (KP835990) and Sc/15/2007 (KP835997). (TIF 5123 kb) Additional file 2: Phylogenetic tree for the H gene nucleotidic sequences. Phylogenetic tree generated with the aligned sequences for the H gene, inferred by Bayesian methods. Sequences for the outgroup taxa were retrieved from NCBI for Pilot Whale Morbillivirus (PWMV [accession number FJ842382]); Porpoise Morbillivirus (PMV [accession number FJ648457]); Phocine Distemper Virus (PDV [accession number KC802221]); Canine Distemper Virus (CDV [accession number AY649446]) and Measles Virus (MV [accession number NC001498]). Three DMV sequences from the 90’s were retrieved from NCBI and included in the trees (Z36778; AJ224705 and AJ608288) along with two sequences from 2007 (HQ829972 and HQ829973). Sequences obtained for the H gene of Portuguese and Galician isolates were also included for animals Sc/31/2009 (KT878652), Sc/290/2014 (KT878651), Sc/221/2012 (KT878650), Sc/55/2012 (KP835989), Sc/11/2013 (KT878649), Dd/302/2012 (KP836001), Dd/191/2013 (KP836005), Sc/257/2011 (KP835985), Sc/53/2012 (KP835993), and Sc/15/2007 (KP835996). (TIF 3936 kb) Additional file: 3 Phylogenetic tree for the N gene nucleotidic sequences. Phylogenetic tree generated with the aligned sequences for the N gene, inferred by Bayesian methods. Sequences for the outgroup taxa were retrieved from NCBI for Pilot Whale Morbillivirus (PWMV [accession number FJ842380]); Porpoise Morbillivirus (PMV [accession number X84739]); Phocine Distemper Virus (PDV [accession number KC802221]); Canine Distemper Virus (CDV [accession number AY649446]) and Measles Virus (MV [accession number NC001498]). Two sequences from 2007 (HQ829972 and HQ829973) and one from the 90’s (AJ608288) were also included in this tree. Sequences obtained for the N gene of Portuguese and Galician isolates were also included for animals Sc/15/2007 (KP835998), Dd/302/2012 (KP836000), Sc/257/2011 (KP835984), Dd/191/2013 (KP836004), Sc/53/2012 (KP835992), Sc/55/2012 (KP835988), Sc/11/2013 (KT878653), Sc/290/2014 (KT878655) and Sc/221/2012 (KT878654). (TIF 4079 kb)

## Abbreviations

BP: *Balaenoptera acutorostrata*
CDV: Canine distemper virus
CEMMA: Coordinadora para o estudo dos mamiferos mariños
CeMV: Cetacean morbillivirus
DD: *Delphinus delphis*
DMV: Dolphin morbillivirus
GM: Globicephala melas
ICNF: Instituto de conservação da natureza e florestas
KB: *Kogia breviceps*
MATBs: Marine animals tissue banks
MMi: *Mesoplodon mirus*
MV: Mealses virus
PDV: Phocine distemper virus
PDV-1: Phocine distemper virus -1
PMV: Porpoise morbillivirus
PP: Posterior probability
PWMV: Pilot whale morbillivirus
RT-PCR: Reverse transcription PCR
RT-qPCR: Reverse transcription quantitative PCR
SC: *Stenella coeruleoalba*
SPVS: Sociedade portuguesa de vida selvagem
TT: *Tursiops truncatus*
