# Supplementary figures and images for: New insight into dolphin morbillivirus phylogeny and epidemiology in the northeast Atlantic: opportunistic study in cetaceans stranded along the Portuguese and Galician coasts
Source: BMC Vet Res. 2016 Aug 26;12(1):176. doi: 10.1186/s12917-016-0795-4 (PMC5002201; doi:10.1186/s12917-016-0795-4)

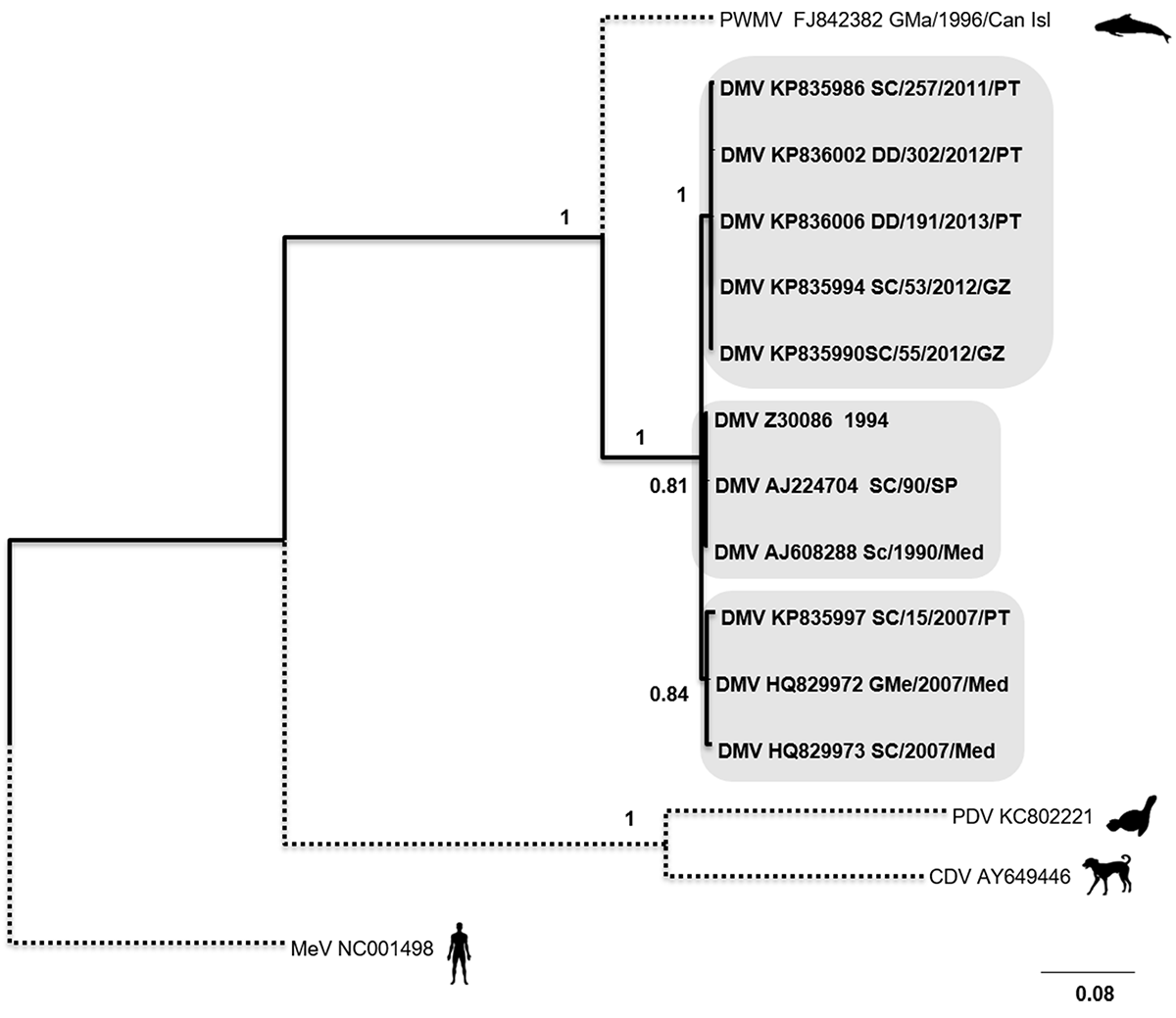

Supplement: Additional file 1: — Phylogenetic tree for the F gene nucleotidic sequences. Phylogenetic tree generated with the aligned sequences for the F gene, inferred by Bayesian methods. Sequences for the outgroup taxa were retrieved from NCBI for Pilot Whale Morbillivirus (PWMV [accession number FJ842382]); Phocine Distemper Virus (PDV [accession number KC802221]); Canine Distemper Virus (CDV [accession number AY649446]) and Measles Virus (MV [accession number NC001498]). Three DMV sequences from the 90’s were retrieved from NCBI and included in the trees (Z30086; AJ224704 and AJ608288) along with two sequences from 2007 (HQ829972 and HQ829973). Sequences obtained for the F gene of Portuguese and Galician isolates were also included for animals Sc/257/2011 (KP835986), Dd/302/2012 (KP836002), Dd/191/2013 (KP836006), Sc/53/2012 (KP835994), Sc/55/2012 (KP835990) and Sc/15/2007 (KP835997). (TIF 5123 kb) [file 12917_2016_795_MOESM1_ESM.tif]

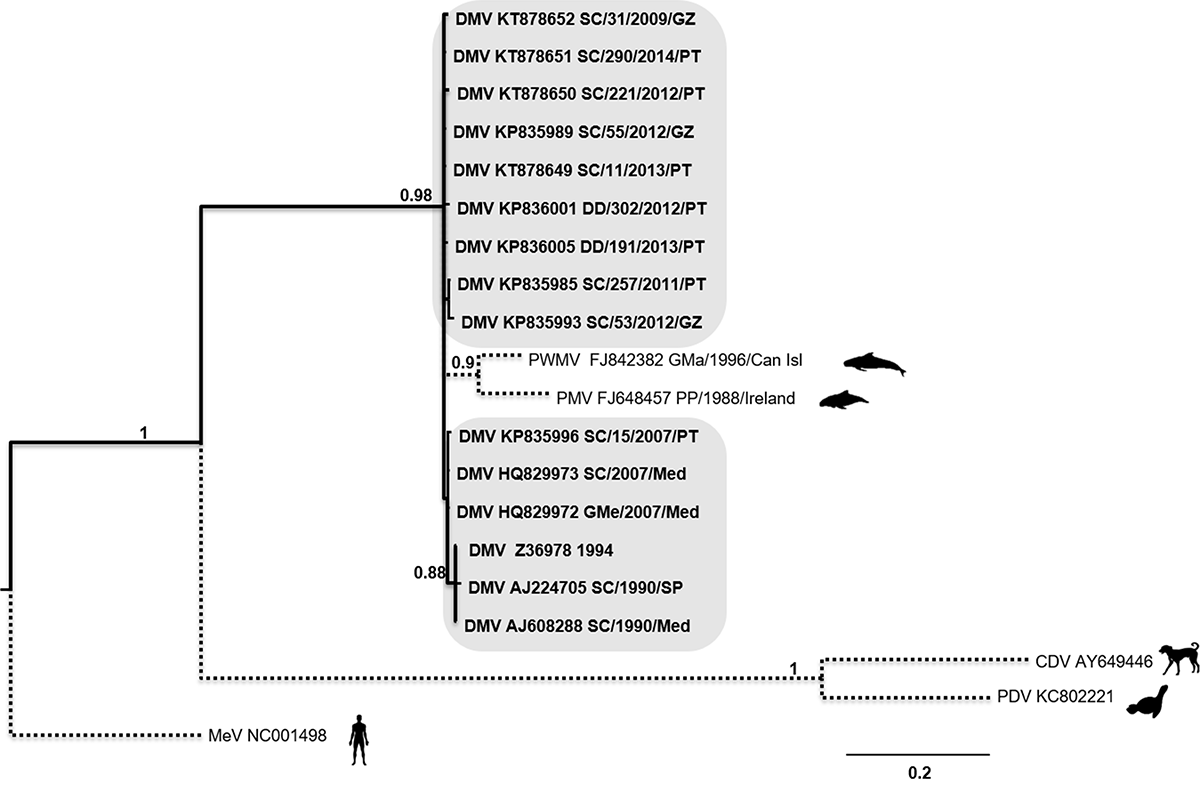

Supplement: Additional file 2: — Phylogenetic tree for the H gene nucleotidic sequences. Phylogenetic tree generated with the aligned sequences for the H gene, inferred by Bayesian methods. Sequences for the outgroup taxa were retrieved from NCBI for Pilot Whale Morbillivirus (PWMV [accession number FJ842382]); Porpoise Morbillivirus (PMV [accession number FJ648457]); Phocine Distemper Virus (PDV [accession number KC802221]); Canine Distemper Virus (CDV [accession number AY649446]) and Measles Virus (MV [accession number NC001498]). Three DMV sequences from the 90’s were retrieved from NCBI and included in the trees (Z36778; AJ224705 and AJ608288) along with two sequences from 2007 (HQ829972 and HQ829973). Sequences obtained for the H gene of Portuguese and Galician isolates were also included for animals Sc/31/2009 (KT878652), Sc/290/2014 (KT878651), Sc/221/2012 (KT878650), Sc/55/2012 (KP835989), Sc/11/2013 (KT878649), Dd/302/2012 (KP836001), Dd/191/2013 (KP836005), Sc/257/2011 (KP835985), Sc/53/2012 (KP835993), and Sc/15/2007 (KP835996). (TIF 3936 kb) [file 12917_2016_795_MOESM2_ESM.tif]

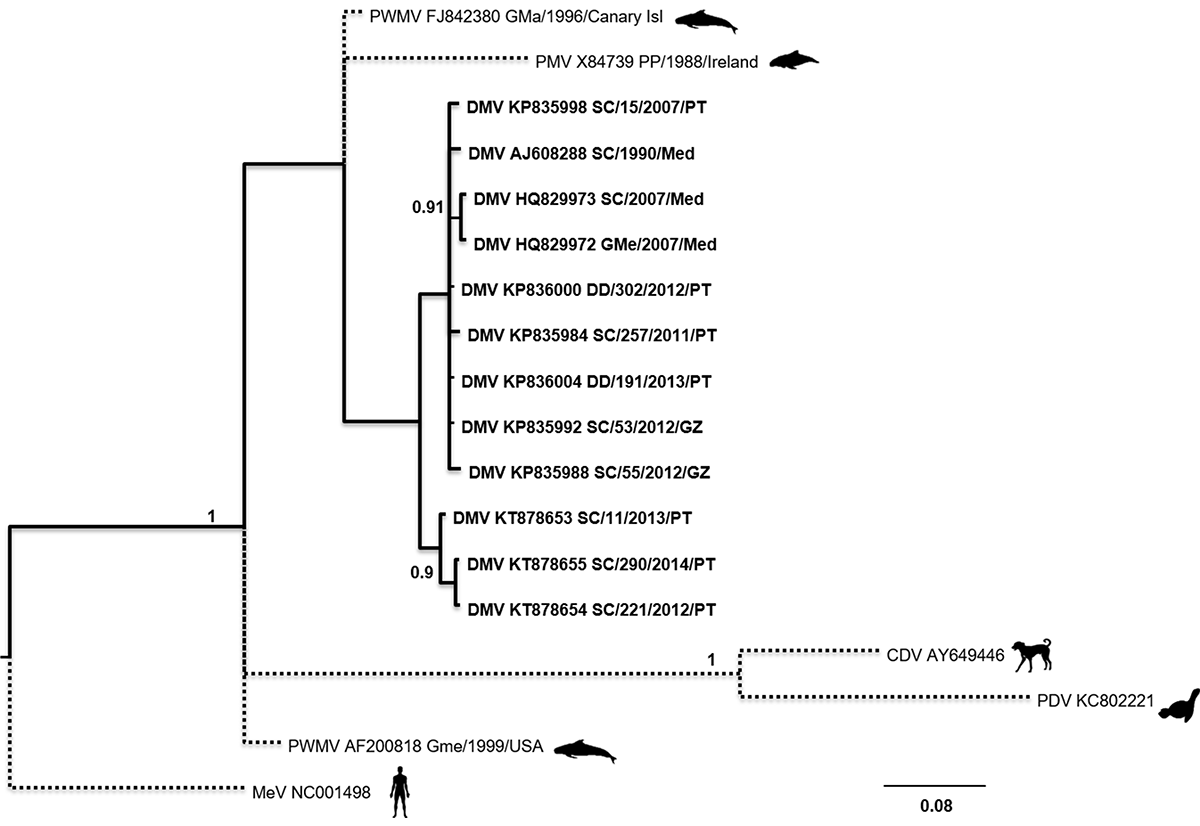

Supplement: Additional file: 3 — Phylogenetic tree for the N gene nucleotidic sequences. Phylogenetic tree generated with the aligned sequences for the N gene, inferred by Bayesian methods. Sequences for the outgroup taxa were retrieved from NCBI for Pilot Whale Morbillivirus (PWMV [accession number FJ842380]); Porpoise Morbillivirus (PMV [accession number X84739]); Phocine Distemper Virus (PDV [accession number KC802221]); Canine Distemper Virus (CDV [accession number AY649446]) and Measles Virus (MV [accession number NC001498]). Two sequences from 2007 (HQ829972 and HQ829973) and one from the 90’s (AJ608288) were also included in this tree. Sequences obtained for the N gene of Portuguese and Galician isolates were also included for animals Sc/15/2007 (KP835998), Dd/302/2012 (KP836000), Sc/257/2011 (KP835984), Dd/191/2013 (KP836004), Sc/53/2012 (KP835992), Sc/55/2012 (KP835988), Sc/11/2013 (KT878653), Sc/290/2014 (KT878655) and Sc/221/2012 (KT878654). (TIF 4079 kb) [file 12917_2016_795_MOESM3_ESM.tif]
